# Green removal of unpleasant volatiles from soapberry (*Sapindus mukorossi*) extracts by two-phase microbial fermentation fortified with pomelo peel waste†

**DOI:** 10.1039/d3ra01858j

**Published:** 2023-04-28

**Authors:** Quoc-Duy Nguyen, Quoc-Duy La, Nhu-Ngoc Nguyen, Thi-Ngoc-Lan Nguyen

**Affiliations:** a Faculty of Environmental and Food Engineering, Nguyen Tat Thanh University Ho Chi Minh City 754000 Vietnam nqduy@ntt.edu.vn

## Abstract

Soapberry (*Sapindus mukorossi* Gaertn) is a popular woody plant in Vietnam, often used as a cleaning product due to its ability to wash, foam and emulsify due to high saponin content. In this study, the performance of fermentation by two microbial strains, namely *Saccharomyces cerevisiae* active dry yeast (ADY) and *Levilactobacillus brevis* lactic acid bacteria (LB) along with the addition of pomelo peel (flavedo) was evaluated during 15 days in terms of sugar removal, antioxidant and antibacterial activities, foaming power, volatile composition, and sensory acceptability. The results showed that the soluble solid content of original extracts experienced a significant decrease from 14.5% to a stable range of 9.4–11.0% until day 15 for all fermented samples, which correlated with a reduction by approximately 60% in reducing sugars (from 12.52 g L^−1^ to 4.77–6.56 g L^−1^). In addition, the saponin content of fermented extracts was in the range of 118.2–145.0 mg L^−1^ while antioxidant activities were extremely reduced after 15 days of fermentation. Increases in pomelo peel imparted fermented extracts with greater antibacterial activity against *Staphylococcus aureus* ATCC 6538, *Proteus mirabilis* ATCC 25933, and *Candida albicans* ATCC 10231, and LB had higher activity than ADY overall. Regarding the volatile profiles, two main compounds in the original extracts, including trilaurin (75.02%) and 1-dodecanoyl-3-myristoyl glycerol (24.85%), were completely removed and replaced by new alkanes, alkenes, alcohols, esters, and organic acids, and particularly d-limonene (86.34–95.31%) upon pomelo addition. Additionally, the foaming ability and stability of fermented extracts were also enhanced and there was clear distinction between fermented and unfermented samples using principal component analysis based on sensory liking data which showed consumers' preference towards fermented samples with a high percentage of pomelo peel.

## Abstract 2

The microbial fermentation by yeast and lactic acid bacteria fortified with pomelo peel waste was performed to purify and improve smell of soapberry extract.

## Graphical abstract

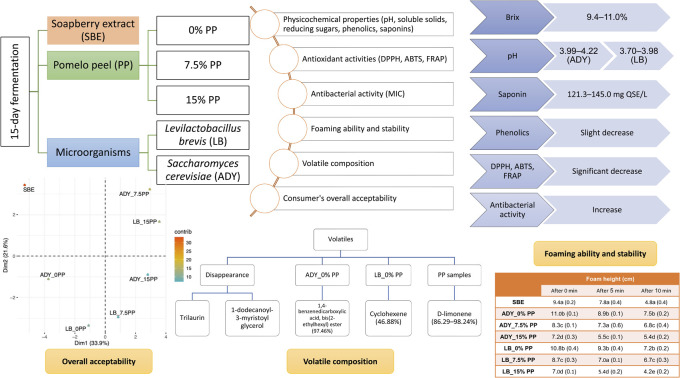

## Introduction

Soapberry (*Sapindus mukorossi* Gaertn) belongs to the family Sapindaceae, commonly known as soapnut, and is a common wild plant in tropical and subtropical climates from Japan to India.^1^ The fruit pericarp contains a high level of saponins (10.1–11.5%) with potential applications as detergents, surfactants, and antibacterial agents in functional cosmetics such as shampoo and cleansers.^2–4^ Saponins are secondary metabolites with a structural characteristic consisting of an aglycone part (triterpene or steroid) linked to mono/disaccharides, typically glucose, arabinose, rhamnose, xylose and galactose *via* ester linkages.^2,3,5^ The majority of plants can only produce one of two types of saponins, namely triterpenoid (dicotyledons) or steroid (monocotyledons).^5^ The applicability of soapberry extracts is limited due to impurities such as sugars (10%), proteins, oils and mucilages native to fruit along with unpleasant odors.^4^ Many studies have been done to purify soapberry extracts such as solvent extraction, use of macroporous resins, ultrafiltration, and foam separation; however, these methods have disadvantages in terms of equipment cost, environmental concerns, and purification efficiency.^6,7^

Fermentation is an age-old biological method that is being viewed as an alternative to other traditional physical purification methods. Fermentation technology can widely change the structure of materials, as microorganisms produce a mixture of extracellular enzymes that disrupt cell membranes.^8^ Various fermentation methods and microorganisms have been used to preserve foods, modifying their organoleptic properties and enhancing their nutritional values.^9^ Among them, lactic acid bacteria and yeast are the most commonly used microorganisms for food fermentation.^10^ Lactic acid bacteria are a group of Gram-positive bacteria capable of converting carbohydrates into organic acids including propionic, formic, acetic and lactic acids along with various metabolites.^11^ Meanwhile, yeast cells generally consume glucose to produce ethanol accompanied by the main by-products of glycerol, acetic acid, and lactic acid.^12^ In the case of purification of saponin extracts, fermentation emerges as an potential approach capable of removing impurities such as sugars and proteins from the soapberry extract, thereby increasing the saponin purity, and improving the surface properties of the extract.^3,6^ Foaming enhancement, microbial inhibition, softening and whitening effect, and freckle removal are just few of the many beneficial effects observed after using its purified saponin solutions.^7^ It is reported that fermentation broth by lactic acid bacteria has been shown to benefit skin health in a number of ways; for instance, lipoteichoic acid from *Lactobacillus plantarum* can suppress melanogenesis, and lactic acid can aid in depigmentation and the reduction of skin wrinkles.^13^

However, there has been little information on the application of other microorganisms into the purification of soapberry extracts and alteration of volatile composition; for example, yeast fermentation has been used in few studies to purify soapberry extract.^3,6^ Therefore, the objectives of the present study were to perform fermentation as green methods for removal of sugars and other undesirable components present in the fruit extract using active dry yeast *Saccharomyces cerevisiae* (ADY) and lactic acid bacteria *Levibactobacillus brevis* (LB). In addition, the valorization of pomelo peel wastes was conducted to improve the unpleasant smell, stickiness, antibacterial ability and foaming power, thereby making the extract easy to apply in other cosmetic products. The fermentation broth was analyzed for changes of physicochemical parameters (pH, soluble solids, reducing sugars, phenolics, saponins), antioxidant activities (DPPH, ABTS, FRAP), microbiological qualities (microorganism density and minimum inhibitory concentration), volatile composition, foaming ability and sensory acceptability. To the best of our knowledge, this is the first study into how microbial fermentation and the addition of pomelo peel waste affect the volatile profiles of soapberry extract. This combination was promising as a practical method for application at the household level.

## Experimental

### Materials and chemicals

Soapberry fruits (mature, brown in color, 2 cm diameter and weight of 15–20 g per fruit) were collected in Kbang (Gia Lai province, Vietnam) in February, 2022 and convectively sun-dried for 24 h to reach the moisture content of 11.29% before storage in PE plastic bags at room temperature, in a dry place. Green pomelo (Tan Trieu cultivar) was grown at Bien Hoa (Dong Nai province, Vietnam). Thermosacc® Dry commercial yeast preparation was supplied by Lallemand Inc. (France) while lactic acid bacteria *Levilactobacillus brevis* UCCLB521 was isolated from kombucha tea at the Microbiology Laboratory (Nguyen Tat Thanh University).

Pathogenic microorganisms, including two Gram-negative bacteria (*Escherichia coli* ATCC 8739 and *Proteus mirabilis* ATCC 25933), two Gram-positive bacteria (*Staphylococcus aureus* ATCC 6538 and *Bacillus cereus* ATCC 11778), and one yeast strain (*Candida albicans* ATCC 10231) were kept frozen in Mueller–Hinton broth (MHB) medium containing 15% v/v glycerol.

Folin–Ciocalteu reagent, 3,5-dinitrosalicylic acid, gallic acid, DPPH, TPTZ, ABTS, and Trolox were purchased from Sigma-Aldrich (Singapore). Mueller–Hinton agar (MHA), Mueller–Hinton broth (MHB), De Man, Rogosa and Sharpe (MRS) agar, and yeast extract were purchased from Hi-Media Laboratory (Mumbai, India). Other chemicals were of analytical grade.

### Preparation and fermentation of soapberry extracts fortified with pomelo peels

The dried soapberry was ground and sifted through a 40-mesh sieve, followed by extraction with distilled water at a material : solvent ratio of 1 : 5 (g mL^−1^) for 2 h. After extraction, the mixture was cloth filtered and centrifuged at 1220*g* for 10 min using a PLC-05 centrifuge (Gemmy Industrial Corp., Taiwan). Subsequently, the clear extracts were pasteurized at 80 °C for 2 min and then rapidly cooled on an ice bath to room temperature. The green pomelo peel (flavedo) was peeled off the white part (albedo), and ground before the addition of water (mass ratio of 1 : 1) and pasteurization using the same procedure described above. Pomelo peel was added to original soapberry extract (SBE) at two levels of 7.5% (7.5% PP) and 15% (15% PP) prior to inoculation of activated yeast (ADY) and *Levilactobacillus brevis* (LB) at 10% inoculum size. The fermentation was conducted under static conditions at room temperature for 15 days and analyzed at day 1, 3, 5, 10, and 15 for the content of total saponins, total phenolics, antioxidant activities and some other physicochemical parameters (reducing sugars, pH, soluble solids) in comparison with control samples (0% PP). In addition, fermented soapberry extracts after 15 days fermentation were evaluated for foaming ability and foam stability, antibacterial activity, volatile composition, and sensory attribute.

### Analysis

#### Total phenolic content

The total phenolic content was performed according to the Folin–Ciocalteu method described according to ISO 14502-1:2005 (ref. 14) based on the reaction of antioxidants with Folin–Ciocalteu reagent in an alkaline medium to form blue chromophore with maximum absorption at 765 nm. The phenolic content was calculated based on the gallic acid standard curve and expressed in mg gallic acid equivalent per liter of extracts (mg GAE per L).

#### Total saponin content

The vanillin–sulfuric acid method was used to quantify the total saponin content as described in the literature.^15^ Briefly, 0.5 mL sample aliquot was added to a test tube containing 0.5 mL vanillin (8% w/v prepared in methanol) and 5 mL sulfuric acid (78% w/v diluted in water) and incubated for 15 min. The absorbance of the sample was measured at 560 nm using a spectrophotometer and total saponin content in the extract was calculated based on a calibration curve using Quillaja saponin standard and expressed in mg Quillaja saponin equivalent per liter of extracts (mg QSE per L).

#### pH, total soluble solids, and reducing sugar content

pH and total soluble solid (°Brix) were measured using the HI 2211-02 pH meter (Hanna Instruments, Romania) and the Master-53M hand-held refractometer (Atago Ltd, Japan), respectively. Reducing sugar content expressed as g glucose per liter was spectrophotometrically determined based on the chromophore from the reaction of reducing sugar and DNS reagent at boiling conditions.^16^ In addition, based on the initial and post-fermentation glucose content, the substrate consumption rate (*Q*_S_, g L^−1^ h^−1^) was calculated.

#### Antioxidant activity – DPPH assay

Antioxidant activity was evaluated through DPPH free radical scavenging capacity based on the purple color change of DPPH solution (0.6 mM) measured at 515 nm upon reaction with antioxidants.^17^ The antioxidant activity of DPPH was calculated against the Trolox calibration curve and expressed in mg Trolox equivalent per liter of extracts (mg TE per L).

#### Antioxidant activity – ABTS assay

ABTS free radical scavenging activity was carried out based on the discoloration of ABTS (7.4 mM) solution measured at 734 nm upon reaction with the antioxidant.^18^ The ABTS cationic radical scavenging activity was calculated against the Trolox calibration curve and expressed in mg Trolox equivalent per liter of extracts (mg TE per L).

#### Antioxidant activity – FRAP assay

Ferric reducing antioxidant power (FRAP) was determined according to Arriola *et al.*^19^ based on the chromophores formed between the working reagents (mixture of 0.3 M acetate buffer at pH 3.6, 0.01 M TPTZ prepared in 0.04 M HCl, and 0.02 M FeCl_3_·6H_2_O solution in a volumetric ratio of 10 : 1 : 1) with antioxidants. Ferric reducing antioxidant activity was calculated against the Trolox calibration curve and expressed in mg Trolox equivalent per liter of extracts (mg TE per L).

#### Microbial enumeration

To determine the density of lactic acid bacteria and yeast, 0.1 mL of diluents was poured on YPDA (yeast extract 5.0 g L^−1^, glucose 20.0 g L^−1^, peptone 5.0 g L^−1^, and agar 20.0 g L^−1^) and MRS agar plates, respectively. The agar plates were then incubated for 48 h at 30 °C and colonies were counted to determine the bacterial density expressed in log number of colony-forming units per milliliter of extract (log CFU mL^−1^).

#### Antibacterial activities – MIC

MIC (minimum inhibitory concentration) is the minimum concentration to inhibit pathogenic microorganisms.^20^ For MIC determination, sample aliquots (100 μL) were serially diluted (dilution factor of 2) using 0.9% NaCl solution in a 96-well plate before adding MHB medium (50 μL) and microbial cultures (50 μL) at a concentration of 10^8^ CFU mL^−1^. After aerobic incubation (37 °C, 24 h), the MIC was verified as the concentration (μL of extract per mL) with no turbidity as indicative of microbial growth.

#### Volatile profiles by GC-MS

The volatile composition and contents were analyzed by gas chromatography coupled mass spectrometry (GC-MS). Briefly, sample aliquot (2 mL) was shaken with 2 mL of *n*-hexane, followed by centrifugation at 5000 rpm for 10 min. The supernatant layer was used for analysis on a GC-MS system (SCION SQ 456-GC/SCION SQ select, SCION, USA) with a silica fused capillary 30 m long column (Rxi-5ms, RESTEK, USA) with particle diameter of 0.25 mm and a film thickness of 0.25 μm using helium as carrier gas. The temperature program was set as follows: the oven temperature was set to 50 °C for 1 min, then increased by 30 °C min^−1^ to 80 °C and further increased by 10 °C min^−1^ to 230 °C, hold for 2 minutes and then increase 25 °C min^−1^ to 280 °C, hold for 8 min. Other fixed parameters were flow rate of 1 mL min^−1^, injection volume of 2 μL, scanning wavelength range of 1–3345 *m*/*z*, ionization voltage of 70 eV, and ionization source temperature of 250 °C.

The volatile compounds were identified by comparing their mass spectra with the mass spectral library of National Institute of Standards and Technology (NIST) (version 2.2, 2014) and by matching their calculated retention indices with the retention indices found in the library. Subsequently, semiquantification was performed to determine their relative concentrations in percent using the peak area of the internal standard (1,3-dichlorobenzene).

#### Foaming ability and stability

The foaming ability and foam stability were determined according to the tube shaking method.^21^ Briefly, 5 mL of the test solution was transferred into a 2 × 18 cm cylindrical test tube before vigorously shaking at a shaking amplitude of 5 cm and a frequency of 3 Hz. Foam height (cm) was recorded at 0, 5 and 10 min to evaluate foam strength over time.

#### Sensory evaluation

The overall acceptability of seven soapberry samples was evaluated by sensory panelists of 30 participants using a five-point hedonic scale (from 1 = ‘extremely dislike’ to 5 = ‘extremely like’) based on sensory attributes, namely smell and stickiness. All participants were students at our institution between the ages of 18 and 25, with women comprising 63%. The participants were not subject to the training session but the repeated contact with original soapberry extracts for one month before the actual sensory evaluation. In addition, the panelists were asked to sign the consent form following ASTM E3314-21 – Standard Guide for Protection of Respondents and Informed Consent for Sensory Evaluation Studies and IFST Guidelines for Ethical and Professional Practices for the Sensory Analysis of Foods. Seven samples (20 mL each) were prepared in covered plastic cups coded with three digits and refrigerated at 5 °C before being randomly presented to panelists using a Latin square design.^22^

#### Statistical analysis

All statistical techniques, including normality test (Shapiro–Wilk's test), homoscedasticity of variances (Levene's test), one-way ANOVA, and *post hoc* Tukey test, were performed at 5% significance level using R version 4.1.2.^23^ Triplicates were used for all tests and measurements. Principal Component Analysis (PCA) based on sensory data was also computed using the FactoMineR package.^24,25^

## Results and discussion

### pH, soluble solid, reducing sugar content, and microbial densities

**Fig. 1:**
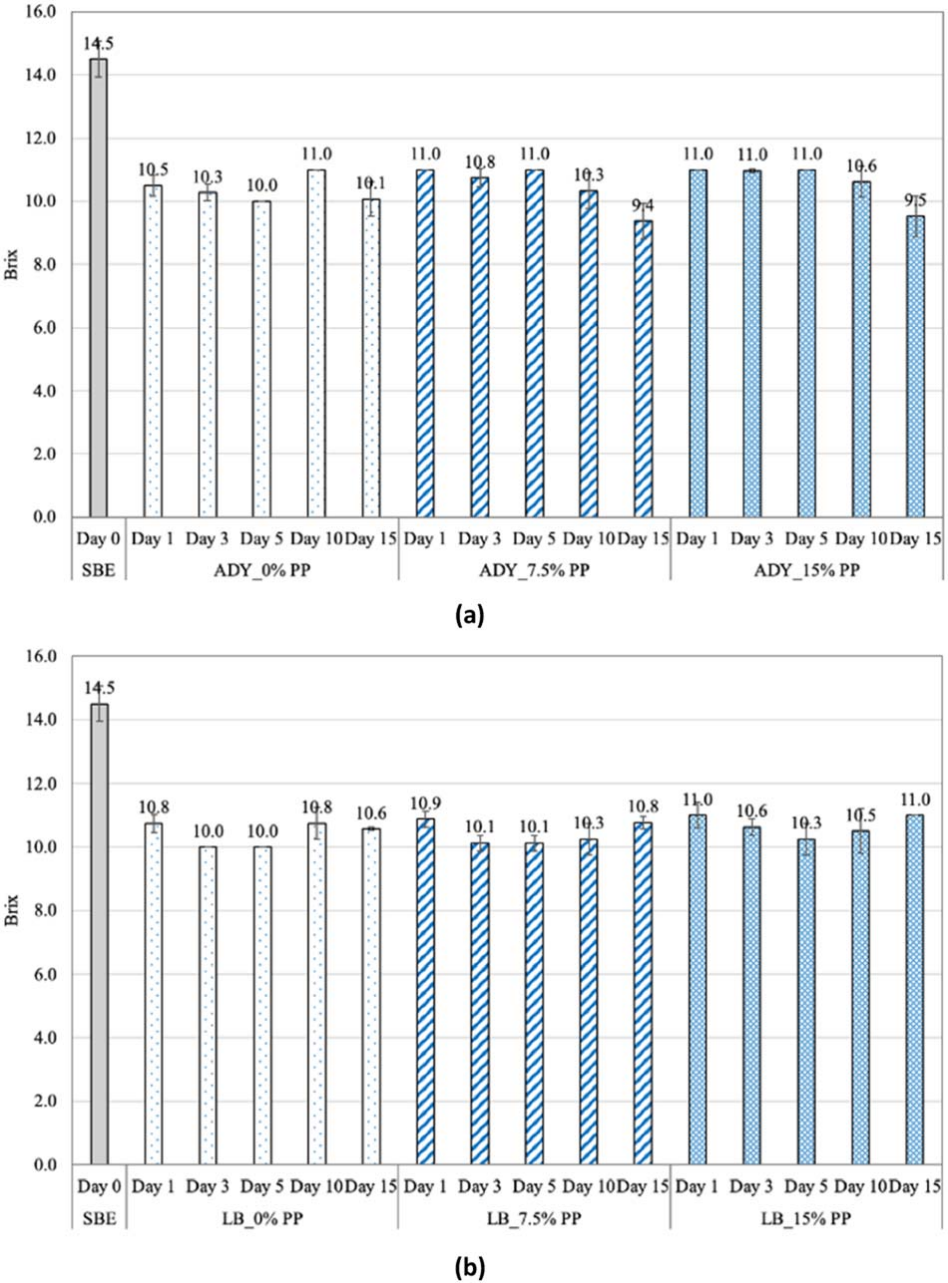
Changes in total soluble solids (°Brix) of soapberry extracts fortified with 0%, 7.5% and 15% pomelo peels (PP) during 15 days fermentation by (a) active dry yeast *Saccharomyces cerevisae* (ADY) and (b) lactic acid bacteria *Levilactobacillus brevis* (LB).

**Fig. 2:**
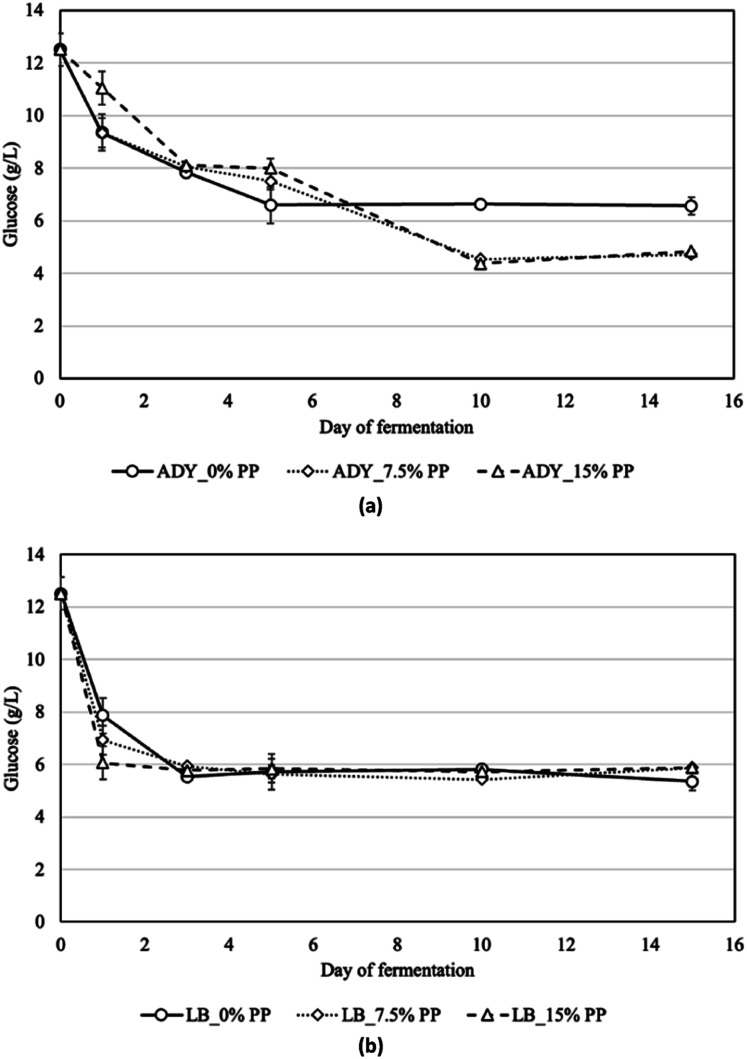
Changes in reducing sugar content (g glucose per L) of soapberry extracts fortified with 0%, 7.5% and 15% pomelo peels (PP) during 15 days fermentation by (a) active dry yeast *Saccharomyces cerevisae* (ADY) and (b) lactic acid bacteria *Levilactobacillus brevis* (LB).

**Fig. 3:**
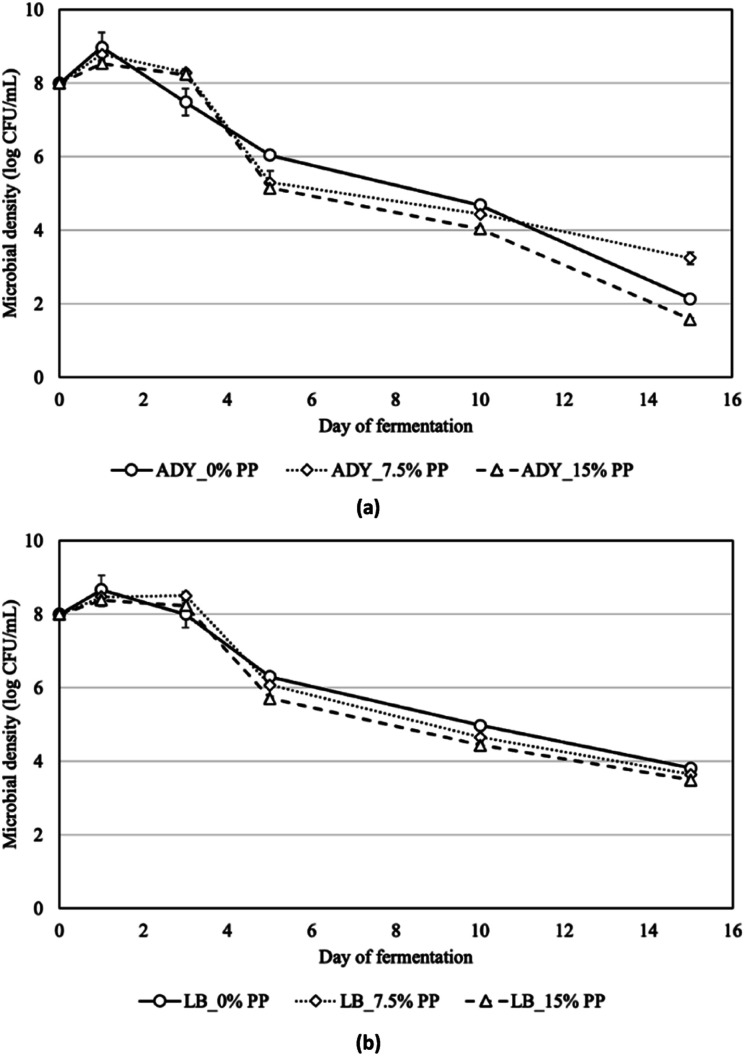
Changes in microbial density (log CFU mL^−1^) of soapberry extracts fortified with 0%, 7.5% and 15% pomelo peels (PP) during 15 days fermentation by (a) active dry yeast *Saccharomyces cerevisae* (ADY) and (b) lactic acid bacteria *Levilactobacillus brevis* (LB).

The changes of soluble solid content (°Brix) and reducing sugar of soapberry extract with and without the addition of pomelo peel during fermentation with commercial yeast *Saccharomyces cerevisiae* and lactic acid bacteria *Levibactobacillus brevis* are presented in Fig. 1 and 2. In general, the soluble solid content of the samples decreased sharply after one day of fermentation, from 14.5% and remained stable until day 15, being 9.4–11.0% for all samples. This result is similar to the finding of Heng *et al.*^6^ who presented the decrease in total soluble solid from 18.29% to 15.30% after yeast fermentation. It is noticeable that for ADY, although the sugar depletion was slower at the first three days in the samples fortified with pomelo peel (Table 1), these values were significantly lower than the control for the last five days of fermentation. This can be attributed to the inhibitory effects of pomelo peel, particularly some native essential oils and antibacterial compounds, at the early stage of fermentation which induced the adaptation of yeast after the exposure to these substances. After acclimatization to the addition of pomelo, the pomelo peel did not interfere much with fermentation because microorganisms could use the pomelo peel as a carbon source during fermentation.^26,27^ This can be concluded by observing the changes in microbial densities during fermentation upon the addition of pomelo peel (Fig. 3). On the first day of fermentation, the microbial counts of ADY and LB appeared to decrease, compared to the control samples. In general, the microbial cell densities of ADY and LB both grew rapidly during the first 3 days of fermentation, 7.48–8.24 (log CFU mL^−1^) for ADY and 8.00–8.50 (log CFU mL^−1^) for LB. Agricultural wastes such as fruit peels have been utilized as carbon source for microbial fermentation by lactic acid bacteria in the study of Parra-Matadamas *et al.*^26^ who concluded that microbial growth was improved upon addition of 1% pomelo peel into fermentation medium, as illustrated by the increase in microbial density from 7.96 log CFU mL^−1^ to 9.02 log CFU mL^−1^.

**Table 1:** Sugar assimilation rate (*Q*_S_, g L^−1^ h^−1^) of soapberry extracts fortified with 0%, 7.5% and 15% pomelo peels (PP) during 15 days fermentation by active dry yeast *Saccharomyces cerevisae* (ADY) and lactic acid bacteria *Levilactobacillus brevis* (LB)

	Day at the end of stationary phase	*Q*_S_ (g L^−1^ h^−1^)
ADY_0% PP	5	0.0494
ADY_7.5% PP	10	0.0332
ADY_15% PP	10	0.0340
LB_0% PP	3	0.0971
LB_7.5% PP	3	0.0915
LB_15% PP	3	0.0937

On the other hand, the sugar content of LB samples showed no differences for the entire process, except for day 1, as illustrated by the fast sugar consumption (*Q*_S_ > 0.09, Table 1). It is common in food fermentation that sugar molecules were assimilated as substrate, resulting in soluble solid depletion^28^ and the production of various metabolites, such as carbonic gas, ethanol and other by-products.^29^ However, there is a difference in the relationship of sugar and °Brix values in soapberry extracts; in which, the high °Brix level of soapberry extracts (14.5%) in this study was not mainly due to sugar. Although sugar decreased sharply but °Brix decreased insignificantly because soluble solid content is inclusive of saponin which are not consumed by microorganisms. Heng *et al.*^6^ also concluded that the fermentation by yeast increased the saponin purity by 75.5%.

**Fig. 4:**
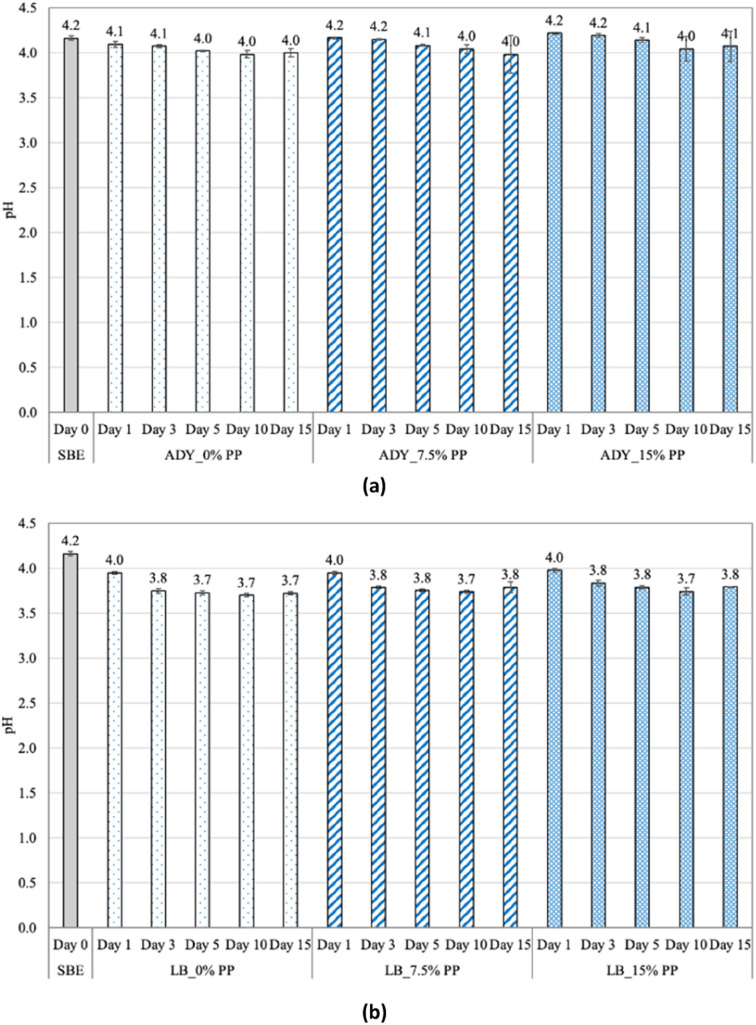
Changes in pH of soapberry extracts fortified with 0%, 7.5% and 15% pomelo peels (PP) during 15 days fermentation by (a) active dry yeast *Saccharomyces cerevisae* (ADY) and (b) lactic acid bacteria *Levilactobacillus brevis* (LB).

Fig. 4 showed that pH values of fermentation broths exhibited insignificant changes for both microorganisms used, ranging from 3.99–4.22 and 3.70–3.98 for ADY and LB, respectively. However, the pH of the fermentation broth by LB was typically lower than that of ADY, which could be explained by the fact that during metabolism, LB mainly converts carbohydrates into lactic acid and organic substances, creating a low pH environment.^30^

### Saponin

**Fig. 5:**
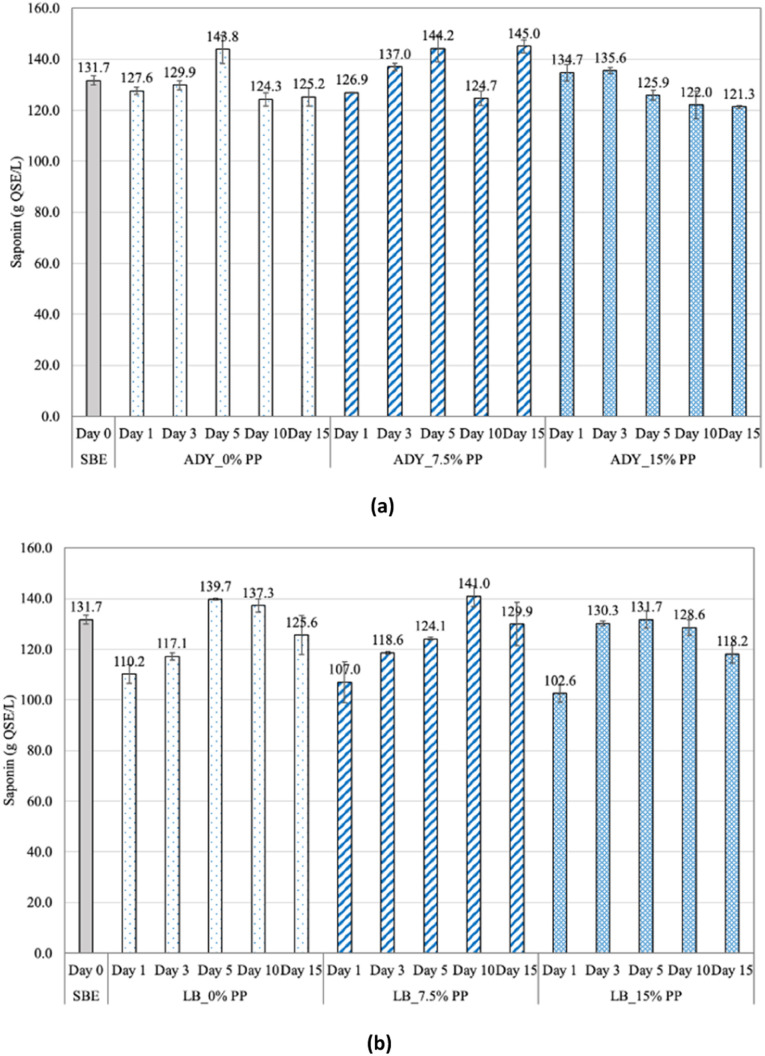
Changes in saponin content (g QSE per L) of soapberry extracts fortified with 0%, 7.5% and 15% pomelo peels (PP) during 15 days fermentation by (a) active dry yeast *Saccharomyces cerevisae* (ADY) and (b) lactic acid bacteria *Levilactobacillus brevis* (LB).

Saponin is a natural surfactant that has many applications in the cosmetic and pharmaceutical fields.^1,3^ The changes of saponin content in fermented soapberry extracts during fermentation are shown in Fig. 5. In general, for ADY, saponin contents remained relatively constant throughout the fermentation regardless of pomelo peel addition and were in the range of 121.3–145.0 mg QSE per L. Similar findings were found in the studies of Chen *et al.*^3^ and Heng *et al.*,^6^ where the saponin content dropped from 38.86 g L^−1^ to 37.72 g L^−1^ and from 18.29% to 15.30% at the end of fermentation by yeast, respectively. Possible explanations for this finding include the fact that the microorganisms used have little effect on saponins.^3^ However, the reduction in saponins by about 23% was observed in LB samples on the first day of fermentation, followed by a gradual increase in the remaining days. This reduction can be ascribed to the metabolites, mainly organic acids, produced during the metabolism of lactic acid bacteria. According to Kim *et al.*,^31^ among the three organic acids (ascorbic acid, citric acid, and malic acid), the impregnation pre-treatment of malic acid resulted in a slight decrease of total saponin content. Another explanation is the adsorption of saponin and other compounds into non-viable lactic acid bacteria and yeast cells depositing at the bottom of fermentation broth.^32,33^ In addition, the increase in saponin content at the remaining days of fermentation could be explained by the diffusion of native saponins present in pomelo peel after long maceration in soapberry extract.^27^

### Phenolic and antioxidant activities

Natural preparations rich in physiologically active substances are increasingly being used in cosmetic products for their antioxidant, anti-inflammatory, anti-aging, antibacterial, and photoprotective effects.^34^ Therefore, the changes of phenolic content and antioxidant activity of fermented soapberry extract were evaluated during 15 days of fermentation and are presented in Table 2. Overall, total phenolic content was relatively unchanged after 15 days fermentation in all samples with and without pomelo peel regardless of microorganisms; however, radical scavenging activity was reduced by a factor of two for both DPPH and ABTS, and by a factor of eight for FRAP. This may be the result of the heat, acid and alcohol produced by fermentation and the hydrolysis of sugars that facilitated the solubilization of phenolics^27^ and the fermentation caused chemical modification of compounds by precipitation, protein binding or adsorption by microorganisms.^35,36^

**Table 2:** Changes in total phenolics (mg GAE per L) and antioxidant activities (mg TE per L) of soapberry extracts fortified with 0%, 7.5% and 15% pomelo peels (PP) during 15 days fermentation by active dry yeast *Saccharomyces cerevisae* (ADY) and lactic acid bacteria *Levilactobacillus brevis* (LB)^a^

	Day of fermentation					
	0	1	3	5	10	15
**Phenolics (mg GAE per L)**						
ADY_0% PP	1850.23a (39.58)	1949.08d (34.07)	2069.24e (26.22)	1718.44b (17.44)	2340.59f (30.21)	1710.68a (45.16)
ADY_7.5% PP		1966.50c (58.24)	2206.85d (16.45)	1683.55b (32.89)	2337.68e (61.67)	1718.44a (100.88)
ADY_15% PP		1999.47d (75.89)	2076.03d (4.11)	1517.84b (28.78)	2256.28e (45.23)	1609.90a (35.05)
LB_0% PP		2009.16d (74.01)	1404.45b (24.67)	1937.45c (17.76)	2323.14e (51.35)	1702.45a (50.33)
LB_7.5% PP		2012.07d (64.66)	1449.03b (38.71)	1946.65d (48.73)	2259.18e (55.47)	1602.15a (20.96)
LB_15% PP		2084.75d (16.45)	1532.37a (26.65)	1951.02c (41.11)	2462.69e (15.38)	1577.43a (57.63)

**DPPH (mg TE per L)**						
ADY_0% PP	2402.30a (75.32)	2422.99c (58.53)	2478.54b (88.86)	1778.88a (54.77)	1693.64a (220.77)	1354.98a (87.52)
ADY_7.5% PP		2278.14b (29.26)	2310.98b (88.86)	1394.41a (30.78)	1742.61a (253.17)	1365.02a (127.77)
ADY_15% PP		2040.18c (73.16)	2541.37d (29.62)	1176.79a (30.78)	1470.58b (46.16)	1043.77a (42.59)
LB_0% PP		3010.48e (115.24)	2164.36c (88.86)	2667.50d (76.94)	1884.06b (46.16)	1475.44a (72.39)
LB_7.5% PP		2725.29d (88.80)	2300.51c (44.43)	3360.27e (228.59)	1916.70b (21.76)	863.07a (237.28)
LB_15% PP		2820.35d (31.12)	2080.58b (29.62)	3505.35e (276.99)	1949.35b (76.94)	812.88a (109.97)

**ABTS (mg TE per L)**						
ADY_0% PP	5588.80a (126.20)	5430.79c (184.30)	2134.10a (66.06)	4101.04b (131.44)	3857.45b (46.8)	2415.56a (132.79)
ADY_7.5% PP		4312.22d (107.51)	4061.08c (181.68)	3729.27c (43.81)	3316.89b (<0.001)	2071.26a (147.55)
ADY_15% PP		5018.11e (122.87)	4154.51d (115.61)	3440.11c (102.23)	3030.07b (93.61)	2290.36a (44.26)
LB_0% PP		4758.96d (44.14)	1953.08a (234.64)	3108.46b (108.57)	3919.97c (108.84)	2224.28a (12.05)
LB_7.5% PP		4727.77c (90.70)	2677.16a (1813.71)	3375.33b (103.65)	3956.74b(31.20)	1810.43a (103.28)
LB_15% PP		4921.96c (179.40)	1836.29a (516.11)	3327.80b (139.58)	3589.01b (55.53)	1946.06a (118.04)

**FRAP (mg TE per L)**						
ADY_0% PP	3279.30a (43.90)	1823.10d (57.56)	1423.83b (35.53)	1564.35c (61.67)	1660.29c (16.45)	468.32a (17.44)
ADY_7.5% PP		1826.97d (38.71)	1456.78b (32.89)	1573.07c (74.01)	1663.20c (12.33)	389.82a (4.11)
ADY_15% PP		1796.93d (28.78)	1347.76c (40.39)	1215.48b (20.56)	1372.47c (30.58)	408.23a (23.50)
LB_0% PP		1663.20d (4.11)	976.12b (26.22)	1535.28c (20.56)	1511.05c (38.71)	552.63a (20.56)
LB_7.5% PP		1669.01d (28.78)	1011.00b (26.86)	1730.06d (8.22)	1576.95c (55.87)	436.34a (12.33)
LB_15% PP		1706.81d (8.22)	1014.88b (20.96)	1622.50d (53.45)	1551.27c (34.36)	407.26a (4.11)

a The results were presented as mean (standard deviation) of triplicates and different letters in the same rows indicate that the mean values were significantly different at 95% confidence level.

It is also concluded that the slight increase in phenolics during the first five days can be explained by the alterations as a result of plant cell wall disruption, allowing the phenolic compounds to diffuse out of the pomelo peel.^27^ It is believed that the active compounds such as antioxidants are present at higher concentration in citrus peel than juice.^37^ During fermentation, microorganisms release enzymes, such as esterases, reductases and decarboxylases that alter the integrity of the cell membranes and hydrolyze the ester bonds of phenol polymers and glycosides.^38^ In addition, due to the use of two-phase fermentation, the fluctuation in the antioxidant contents and activities are likely the result of complex relationship of bioconversion by microbial metabolism, antioxidant diffusion from pomelo peel, and their adsorption into pomelo peel.

### Antibacterial activity, foaming activity and stability

MIC for pathogenic organisms including bacteria and fungi were performed on the pre- and post-fermentation soapberry extracts with the addition of pomelo peel and are presented in Table 3. For Gram-positive bacteria, the fermentation as well as the addition of pomelo peel significantly changed the antibacterial activity compared with the non-fermented sample (SBE); particularly, the improved inhibition against *Staphylococcus aureus* ATCC 6538. Bacterial growth can be stifled by lactic acid and other fermentation byproducts, particularly bacteriocins produced by lactic acid bacteria.^39^ Furthermore, the essential oils derived from pomelo flavedo also contributes to the increased antibacterial activity of soapberry extracts, as illustrated by the higher activity upon increasing the percentage of pomelo peel in the fermentation medium.

**Table 3:** MIC of soapberry extracts fortified with 0%, 7.5% and 15% pomelo peels (PP) after 15 days fermentation by active dry yeast *Saccharomyces cerevisae* (ADY) and lactic acid bacteria *Levilactobacillus brevis* (LB)^a^^,^^b^

	MIC (μL mL^−1^)				
	Gram-positive		Gram-negative		Fungi
	Sta	Bac	Esc	Pro	Can
SBE	250	500	250	500	500
ADY_0% PP	500	500	500	500	500
ADY_7.5% PP	62.5	500	250	125	15.6
ADY_15% PP	250	500	250	500	250
LB_0% PP	125	500	125	62.5	250
LB_7.5% PP	125	250	250	250	15.6
LB_15% PP	62.5	500	125	125	125

a Pathogen abbreviation: Sta (*Staphylococcus aureus* ATCC 6538), Bac (*Bacillus cereus* ATCC 11778), Esc (*Escherichia coli* ATCC 8739), Pro (*Proteus mirabilis* ATCC 25933), Can (*Candida albicans* ATCC 10231).

b SBE – original soapberry extracts.

tSaponin is a natural surfactant that has hydrophilic sugar molecules and a hydrophobic sapogenin moiety.^40^ Foaming ability is one of the important properties of saponins,^5^ so determining the foaming ability of saponins present in extracts before and after fermentation is also very important. The foaming ability and foam stability over time of the fermented soapberry extract were shown in Table 4. It is noteworthy that the foaming ability of fermented soapberry extracts without the addition of pomelo peel (10.8–11.0 cm) was improved compared with SBE (9.4 cm) whereas increasing the pomelo peel resulted in the attenuation of foaming ability. However, in terms of the foaming stability within 10 min, the fermented extracts with pomelo peel exerted more stable foam than unfermented samples. In general, there was no difference in the foaming ability and stability between two microorganisms investigated. This result is in accordance with the study of Chen *et al.*,^3^ which indicated that fermentation not only improved saponin purity but also significantly promoted foam stability. Wei *et al.*^7^ also concluded that fermented soapberry extracts showed increase in detergent activity by 11.3% for dirty cloths and 20% for protein and sebum with double antibacterial activity against *Trichophyton rubrum* ATCC 294 and *Candida albicans* ATCC 10231. The increase in foaming capacity may be due to fermentation leading to some bioconversion such as partial hydrolysis of saponins to the corresponding aglycone (sapogenins), which is more active than saponins.^5^ In addition, the removal of components such as proteins, carbohydrates and other components resulting from fermentation increased surface tension, thereby increasing the foaming power.^3,7^

**Table 4:** Foaming activity and stability of soapberry extracts fortified with 0%, 7.5% and 15% pomelo peels (PP) after 15 days fermentation by active dry yeast *Saccharomyces cerevisae* (ADY) and lactic acid bacteria *Levilactobacillus brevis* (LB)^a^^,^^b^

	Foam height (cm)		
	After 0 min	After 5 min	After 10 min
SBE	9.4a (0.2)	7.8a (0.4)	4.8a (0.4)
ADY_0% PP	11.0b (0.1)	8.9b (0.1)	7.5b (0.2)
ADY_7.5% PP	8.3c (0.1)	7.3a (0.6)	6.8c (0.4)
ADY_15% PP	7.2d (0.3)	5.5c (0.1)	5.4d (0.2)
LB_0% PP	10.8b (0.4)	9.3b (0.4)	7.2b (0.2)
LB_7.5% PP	8.7c (0.3)	7.0a (0.1)	6.7c (0.3)
LB_15% PP	7.0d (0.1)	5.4d (0.2)	4.2e (0.2)

a The results were presented as mean (standard deviation) of triplicates and different letters in the same columns indicate that the mean values were significantly different at 95% confidence level.

b SBE – original soapberry extracts.

#### Volatile profiles

The volatile composition of soapberry extracts fermented by ADY and LB were compared with non-fermented soapberry extracts (SBE) and presented in Table 5. The results showed that major volatile compounds in the SBE sample were trilaurin (75.02%) and 1-dodecanoyl-3-myristoyl glycerol (24.85%), which were completely absent in all fermented samples regardless of the addition of pomelo peel. Besides, its is observed in the fermented samples without pomelo that the newly synthesized volatile component, accounting for a large amount, such as 1,4-benzenedicarboxylic acid, bis(2-ethylhexyl) ester (97.46% in ADY_0% PP) apart from a wide range of new alkanes and alkenes, especially 1-octadecanol (45.72% in LB_0% PP). According to some literature, 1.4-benzenedicarboxylic acid, bis(2-ethylhexyl) ester shows slight odor.^41^ In case of fermented samples with the addition of pomelo peel, d-limonene appeared to dominate (86.34–95.31%) and their volatile composition was mainly alkanes, alkenes, alcohols, esters, and organic acids. The predominance of d-limonene in fermented samples was associated with the occurrence of this substance in native pomelo peel,^42^ which plays an important role in improving the aroma of fermented extracts upon the addition of pomelo peel.

**Table 5:** Volatile profiles of soapberry extracts fortified with 0%, 7.5% and 15% pomelo peels (PP) after 15 days fermentation by active dry yeast *Saccharomyces cerevisae* (ADY) and lactic acid bacteria *Levilactobacillus brevis* (LB)^a^

Compounds	MW	*R*_*t*_	RI	SBE	ADY_0% PP	ADY_7.5% PP	ADY_15% PP	LB_0% PP	LB_7.5% PP	LB_15% PP
1-Cyclohexyl-2-propen-1-ol	140	4.45	705							
1-Dodecanoyl-3-myristoyl glycerol	484	26.19	678	24.85						
1-Ethylundecyl methoxyacetate	272	23.27	720			10.21				
1-Octadecanol	270	27.09	755					45.72		
1,4-Benzenedicarboxylic acid, bis(2-ethylhexyl) ester	390	25.65	892		97.46		3.77		1.18	7.13
2-Methyleicosane	296	20.74	705			8.13				
2,6-Octadien-1-ol, 2.7-dimethyl-	154	10.76	740				0.19		0.62	0.16
3,4-Hexanediol, 2,5-dimethyl-	146	3.79	700							
3,9-Epoxypregn-16-ene-14-18-diol-20-one, 7.11-diacetoxy-3-methoxy-	492	26.72	444	0.06						
7-Hexadecenoic acid	254	23.72	734					17.57		
Azafrin methyl ester	440	26.50	444	0.03						
Carveol	152	9.52	667				0.08			
*cis*-Sabinenhydrate	154	8.98	695				0.21		0.3	0.16
Cyclohexanol	136	5.64	751		2.54					
d-Limonene	136	5.65	917			46.87	94.35		95.31	86.34
Heptadecane, 9-hexyl-	324	22.79	727			13.42				
Hexadecane	226	19.16	708			10.9				
Isoauraptene	260	24.00	840				0.26		0.25	0.19
Mono(2-ethylhexyl) phthalate	278	25.00	574	0.04						
*n*-Hexadecanoic acid	256	22.58	770							0.13
Nonadecane	268	21.53	741			10.46				
Oleic acid	282	25.61	677					14.04		
Osthole	244	23.57	798				0.14		0.16	0.12
Tetratetracontane	618	23.69	707							0.1
*trans*-β-Ocimene	136	4.06	812				0.35		0.42	0.25
Tridecanoic acid	214	24.12	670					22.67		
Trilaurin	638	27.26	700	75.02						
α-Phellandrene	136	5.19	756						0.43	0.17
α-Terpineol	154	9.29	803				0.19		0.48	0.17
β-Pinene	136	4.86	854				0.47		0.86	0.47
β-Sitosterol	414	24.30	780							4.61

a Rt – retention time, RI – retention index.

It is known that fermentation can alter the organoleptic properties of foods and other fermented products.^8^ Proteolysis is one of the major biochemical processes in flavor development that occurs during fermentation. Two main pathways are involved in the conversion of free amino acids to volatile compounds, including the reduction catalyzed by lyase and the pathway initiated by aminotransferases.^43^ In addition, lipolysis and fatty acid oxidation in fermented foods are the main sources of flavor compounds, which can be produced by lipase present in relatively high concentrations in *Lactococcus* and *Lactobacillus* species.^44^

### Sensory evaluation

**Fig. 6:**
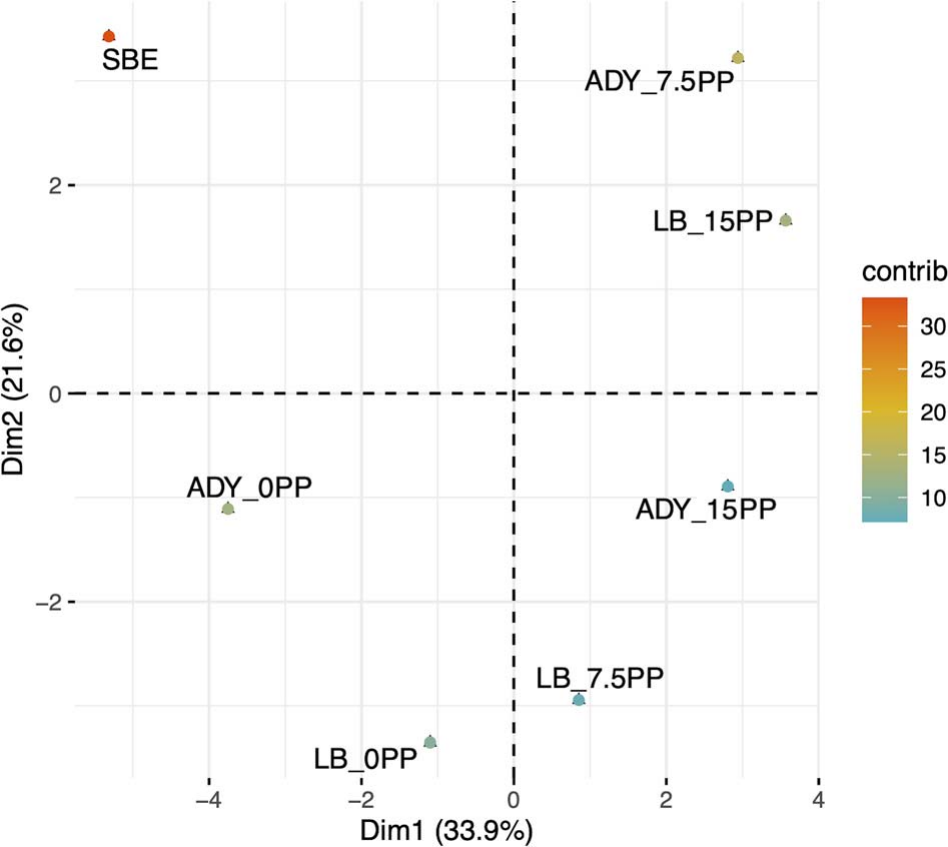
Preference mapping of unfermented (SBE) and fermented soapberry extracts fortified with 0%, 7.5% and 15% pomelo peels (PP) after 15 days fermentation by active dry yeast *Saccharomyces cerevisae* (ADY) and lactic acid bacteria *Levilactobacillus brevis* (LB) in the first two dimensions of PCA plot.

The overall likings of seven soapberry extracts based on five-point hedonic scale are shown in Table 6. It is observed that the fermented samples with the addition of pomelo peel were preferred by consumer, with liking values being 3.13–3.40 (15% PP) as opposed to original extract (2.37). This can be explained by the pleasant aroma of pomelo peel, which is mainly d-limonene, and the lower stickiness resulting from the depletion of sugar content. Furthermore, PCA technique was also applied to discriminate the seven samples and the PCA results presented in Fig. 6 displayed the clustering among non-fermented/fermented samples and with/without the addition of pomelo peel. Specifically, the first and second principal components (PC) accounted for 55.5% of the variation in the liking data. It is noteworthy that SBE was separately located at the upper left quarter of PCA plot with significantly negative impact on PC1, implying its distinct property compared to the other fermented samples. On the other hand, ADY_7.5% PP, ADY_15% PP, and LB_15% PP showed significantly positive contribution on both PC1 and PC2. Other groups could be established as without pomelo peel addition (ADY_0% PP and LB_0% PP) and with pomelo addition (ADY_7.5% PP, ADY_15% PP, LB_7.5% PP, and LB_15% PP). It could be concluded that the fermentation and addition of pomelo peel showed positive effect on the organoleptic properties of original soapberry extracts.

**Table 6:** Mean scores of consumers' overall liking of unfermented (SBE) and fermented soapberry extracts fortified with 0%, 7.5% and 15% pomelo peels (PP) during 15 days fermentation by active dry yeast *Saccharomyces cerevisae* (ADY) and lactic acid bacteria *Levilactobacillus brevis* (LB)^a^

Samples	Liking
SBE	2.37a (1.16)
ADY_0% PP	2.50a (1.17)
ADY_7.5% PP	3.23c (1.04)
ADY_15% PP	3.13bc (0.97)
LB_0% PP	2.63b (1.07)
LB_7.5% PP	2.77b (0.94)
LB_15% PP	3.40c (1.00)

a The results were presented as mean (standard deviation) of triplicates and different letters in the same columns indicate that the mean values were significantly different at 95% confidence level.

## Conclusions

Fermentation of soapberry extract by yeast and bacteria improved saponin purity by removing impurities, mainly sugars, in the original extract. In particular, the combination of fermentation and pomelo flavedo has completely eliminated the unpleasant smell of soapberry extracts and imparted a pleasant scent, mainly d-limonene to the fermented extracts. This is also reflected in the high sensory acceptability of fermented samples and the clear clustering between fermented and unfermented ones. In addition, the foaming ability and antibacterial activity of the extract were also significantly enhanced compared to original soapberry extract.

## Author contributions

Quoc-Duy Nguyen: conceptualization; data curation; investigation; methodology; visualization; writing – original draft; writing – review & editing. Quoc-Duy La: investigation; data curation; writing – original draft. Nhu-Ngoc Nguyen: visualization; data curation; writing – original draft. Thi-Ngoc-Lan Nguyen: conceptualization, data curation.

## Conflicts of interest

There are no conflicts to declare.

## Supplementary Material

RA-013-D3RA01858J-s001
