# Supplementary material for: Green removal of unpleasant volatiles from soapberry (Sapindus mukorossi) extracts by two-phase microbial fermentation fortified with pomelo peel waste
Source: RSC Adv. 2023 Apr 28;13(19):13282–91. doi: 10.1039/d3ra01858j (PMC10142458; doi:10.1039/d3ra01858j)
Supplement: RA-013-D3RA01858J-s001 [file RA-013-D3RA01858J-s001.pdf]

This journal is © The Royal Society of Chemistry 2023

Supplementary data

Table S1. Changes in total soluble solids (°Brix), pH, and reducing sugar content (g glucose/L) of soapberry extracts fortified with 0%, 7.5% và 15% pomelo peels (PP) during 15-day fermentation by active dry yeast *Saccharomyces cerevisae* (ADY) and lactic acid bacteria *Levilactobacillus brevis* (LB).

Table S2. Changes in saponin content (g QSE/L) of soapberry extracts fortified with 0%, 7.5% và 15% pomelo peels (PP) during 15-day fermentation by active dry yeast *Saccharomyces cerevisae* (ADY) and lactic acid bacteria *Levilactobacillus brevis* (LB).

Table S3. Changes in microbial density (log CFU/mL) of soapberry extracts fortified with 0%, 7.5% và 15% pomelo peels (PP) during 15-day fermentation by active dry yeast *Saccharomyces cerevisae* (ADY) and lactic acid bacteria *Levilactobacillus brevis* (LB).

Table S4. Consumers' overall liking scores of unfermented (SBE) and fermented soapberry extracts fortified with 0%, 7.5% và 15% pomelo peels (PP) after 15-day fermentation by (a) active dry yeast *Saccharomyces cerevisae* (ADY) and (b) lactic acid bacteria *Levilactobacillus brevis* (LB).

Table S1. Changes in total soluble solids (°Brix), pH, and reducing sugar content (g glucose/L) of soapberry extracts fortified with 0%, 7.5% và 15% pomelo peels (PP) during 15-day fermentation by active dry yeast *Saccharomyces cerevisae* (ADY) and lactic acid bacteria *Levilactobacillus brevis* (LB).

	Day of fermentation					
	0	1	3	5	10	15
**Brix**						
ADY_0% PP	14.5a (0.6)	10.5b (0.33)	10.3b (0.3)	10.0b (<0.01)	11.0b (<0.01)	10.1b (0.6)
ADY_7.5% PP		11.0b (<0.01)	10.8b (0.3)	11.0b (<0.01)	10.3b (0.6)	9.4a (0.6)
ADY_15% PP		11.0b (<0.01)	11.0b (0.1)	11.0b (<0.01)	10.6b (0.5)	9.5a (0.7)
LB_0% PP		10.8b (0.3)	10.0a (<0.01)	10.0b (<0.01)	10.8b (0.5)	10.6a (0.1)
LB_7.5% PP		10.9b (0.3)	10.1b (0.3)	10.1b (0.3)	10.3b (0.5)	10.8b (0.2)
LB_15% PP		11.0b (0.4)	10.6b (0.3)	10.3b (0.5)	10.5b (0.7)	11.0b (<0.01)
**pH**						
ADY_0% PP	4.17a (0.02)	4.10c (0.03)	4.07c (0.02)	4.02b (0.01)	3.99a (0.04)	4.00a (0.05)
ADY_7.5% PP		4.17a (0.01)	4.15a (0.01)	4.09a (0.01)	4.04a (0.05)	3.99a (0.21)
ADY_15% PP		4.22a (0.01)	4.19a (0.02)	4.14a (0.03)	4.04a (0.14)	4.07a (0.17)
LB_0% PP		3.95c (0.01)	3.75b (0.03)	3.73a (0.02)	3.70a (0.02)	3.73a (0.02)
LB_7.5% PP		3.95b (0.02)	3.79a (0.02)	3.75a (0.01)	3.74a (0.02)	3.77a (0.06)
LB_15% PP		3.98c(0.02)	3.84b (0.03)	3.79a (0.02)	3.75a (0.04)	3.79a (<0.001)
**Glucose (g/L)**						
ADY_0% PP	12.52a (0.62)	9.35b (0.69)	7.83a (0.08)	6.59a (0.68)	6.63a (0.04)	6.56a (0.34)
ADY_7.5% PP		9.33c (0.55)	8.00b (0.04)	7.55b (0.32)	4.55a (0.02)	4.77a (0.11)
ADY_15% PP		11.00c (0.64)	8.11b (0.01)	7.99b (0.38)	4.33a (0.10)	4.88a (0.05)
LB_0% PP		7.88b (0.15)	5.55a (0.10)	5.77a (0.27)	5.88a (0.11)	5.33a (0.27)
LB_7.5% PP		6.99b (0.28)	5.99a (0.18)	5.66a (0.12)	5.44a (0.18)	5.88a (0.28)
LB_15% PP		6.00a (0.02)	5.77a (0.09)	5.88a (0.08)	5.77a (0.07)	5.88a (0.24)

Notes: The results were presented as mean (standard deviation) of triplicates and different letters in the same columns indicate that the mean values were significantly different at 95% confidence level.

Table S2. Changes in saponin content (g QSE/L) of soapberry extracts fortified with 0%, 7.5% và 15% pomelo peels (PP) during 15-day fermentation by active dry yeast *Saccharomyces cerevisae* (ADY) and lactic acid bacteria *Levilactobacillus brevis* (LB).

	Day of fermentation					
	0	1	3	5	10	15
	Saponin (g QSE/L)					
ADY_0% PP	131.73a (1.88)	127.51a (1.48)	129.91a (1.81)	143.81b (5.63)	124.31a (2.57)	125.21a (3.51)
ADY_7.5% PP		126.87a (0.28)	136.91b (1.64)	144.11c (5.17)	124.71a (2.77)	145.01c (2.49)
ADY_15% PP		134.73c (3.33)	135.51c (1.19)	125.91a (1.85)	122.01a (5.45)	121.31a (0.60)
LB_0% PP		110.60a (3.79)	117.11a (1.34)	139.71d (0.37)	137.21d (2.62)	125.61b (7.70)
LB_7.5% PP		106.96a (8.12)	118.61a (0.42)	124.11b (0.65)	140.91c (4.10)	129.91b (8.52)
LB_15% PP		102.59a (3.42)	130.21c (0.94)	131.71c (3.42)	128.51c (3.13)	118.19 (3.55)

Notes: The results were presented as mean (standard deviation) of triplicates and different letters in the same columns indicate that the mean values were significantly different at 95% confidence level.

Table S3. Changes in microbial density (log CFU/mL) of soapberry extracts fortified with 0%, 7.5% và 15% pomelo peels (PP) during 15-day fermentation by active dry yeast *Saccharomyces cerevisae* (ADY) and lactic acid bacteria *Levilactobacillus brevis* (LB).

<table>
<tr><th></th><th colspan="6">Day of fermentation</th></tr>
<tr><th></th><th>0</th><th>1</th><th>3</th><th>5</th><th>10</th><th>15</th></tr>
<tr><td colspan="7">Microbial density (log CFU/mL)</td></tr>
<tr><td>ADY_0% PP</td><td rowspan="6">8.00a (<0.01)</td><td>8.98a (0.39)</td><td>7.48a (0.37)</td><td>6.05a (0.05)</td><td>4.70a (0.01)</td><td>2.14a (<0.01)</td></tr>
<tr><td>ADY_7.5% PP</td><td>8.78a (0.25)</td><td>8.29a (0.10)</td><td>5.30a (0.31)</td><td>4.44a (0.06)</td><td>3.23a (0.16)</td></tr>
<tr><td>ADY_15% PP</td><td>8.16a (0.09)</td><td>8.24a (0.07)</td><td>5.15a (<0.01)</td><td>4.04a (0.06)</td><td>1.57a (0.04)</td></tr>
<tr><td>LB_0% PP</td><td>8.67a (0.42)</td><td>8.00a (<0.01)</td><td>6.30a (0.01)</td><td>4.98a (0.03)</td><td>3.81a (<0.01)</td></tr>
<tr><td>LB_7.5% PP</td><td>8.48a (0.13)</td><td>8.50a (0.28)</td><td>6.07a (0.17)</td><td>4.65a (0.07)</td><td>3.64a (0.02)</td></tr>
<tr><td>LB_15% PP</td><td>8.38a (0.27)</td><td>8.24a (0.34)</td><td>5.71a (0.19)</td><td>4.44a (0.06)</td><td>3.49a (0.01)</td></tr>
</table>

Notes: The results were presented as mean (standard deviation) of triplicates and different letters in the same columns indicate that the mean values were significantly different at 95% confidence level.

Table S4. Consumers' overall liking scores of unfermented (SBE) and fermented soapberry extracts fortified with 0%, 7.5% và 15% pomelo peels (PP) after 15-day fermentation by (a) active dry yeast *Saccharomyces cerevisae* (ADY) and (b) lactic acid bacteria *Levilactobacillus brevis* (LB).

Panelist	SBE	ADY_0% PP	ADY_7.5% PP	ADY_15% PP	LB_0% PP	LB_7.5% PP	LB_15% PP
1	2	3	2	2	3	3	3
2	2	2	3	4	3	4	3
3	2	3	3	4	2	3	3
4	2	2	3	2	2	2	3
5	2	2	3	4	2	2	3
6	5	3	5	5	3	3	5
7	2	2	2	2	2	2	2
8	4	3	4	3	2	2	4
9	3	4	4	4	2	2	2
10	2	2	3	4	3	4	4
11	2	4	4	4	4	4	4
12	4	3	4	2	3	3	3
13	2	2	2	2	2	2	2
14	2	2	5	4	2	3	4
16	3	3	3	3	3	3	3
17	0	0	1	2	0	1	1
18	3	2	3	2	2	3	4
19	5	1	3	2	1	2	3
20	2	5	5	4	4	1	5
21	3	4	5	5	5	5	4
22	2	2	3	2	2	2	3
23	3	3	4	4	4	4	5
24	3	3	3	3	3	3	3
25	3	2	2	3	4	2	4
26	1	1	3	2	1	2	2
27	2	1	3	3	4	3	4
28	1	2	4	3	3	4	4
29	2	3	4	3	3	3	3
30	2	1	2	3	3	3	4

32
